# Defining the link between peripheral neuronal activity and neuropathic pain: observational study protocol to investigate *in vivo* neurophysiological properties of nociceptors in patients with chronic neuropathic pain

**DOI:** 10.1371/journal.pone.0349407

**Published:** 2026-07-16

**Authors:** Andreas C. Themistocleous, Georgios Baskozos, David L. H. Bennet, Jordi Serra

**Affiliations:** 1 Nuffield Department of Clinical Neurosciences, University of Oxford, Oxford, United Kingdom; 2 Department of Clinical Neurophysiology, Kings College Hospital NHS Foundation Trust, Denmark Hill, London, United Kingdom; University of Minho, PORTUGAL

## Abstract

**Introduction:**

Neuropathic pain affects up to 10% of the population and remains difficult to diagnose and treat effectively. Peripheral nociceptor hyperexcitability is thought to drive pain in a substantial subset of patients, yet human studies have not fully defined which nociceptor populations are abnormal or how these abnormalities relate to clinical phenotype, sensory profiling, or genetic mechanisms. Microneurography uniquely enables direct in vivo recording from human C-fibres, offering a mechanistic approach to patient stratification. This observational study investigates the relationships between neuropathic pain, nociceptor excitability, somatosensory phenotype, and genetic variation.

**Methods and analysis:**

We will conduct an observational controlled study at the University of Oxford (2022–2029) following STROBE guidelines. Adults with and without neuropathy or neuropathic pain will undergo deep phenotyping, including questionnaires, neurological examination, quantitative sensory testing, nerve conduction studies, microneurography, skin biopsy, and genetic and transcriptomic analyses. Microneurography recordings will quantify C-fibre axonal properties, excitability, spontaneous activity, and responses to electrical, thermal, and mechanical stimulation. Participants will be stratified by neuropathy grade, neuropathic pain status, somatosensory phenotype, and genetic variants. Primary analyses will compare axonal excitability and related microneurography measures between participants with and without neuropathic pain. Secondary analyses will evaluate the associations between microneurography-derived nociceptor functional profiles and quantitative sensory phenotypes, and will assess the impact of rare ion-channel variants on C-fibre excitability. Multivariable regression, dimensionality reduction, and unsupervised clustering methods will be applied to delineate mechanistically informed patient subgroups.

**Ethics and dissemination:**

Ethical approval was obtained from the South Central – Oxford C Research Ethics Committee (18/SC/0263). All participants will provide informed consent. Findings will be disseminated through peer-reviewed publications, conference presentations, and data-sharing compliant with institutional and funding-body policies.

**Strengths and limitations of this study:**

The study uses microneurography, the only technique that directly records from human nociceptors in vivo, enabling detailed mechanistic assessment of C-fibre function. A deep phenotyping framework, including quantitative sensory testing, skin biopsy, nerve conduction studies, and genetic analysis, allows multidomain integration of clinical, neurophysiological, and molecular data. Classification of C-fibre subtypes using activity-dependent slowing provides a reproducible method to distinguish functional nociceptor populations. Extended microneurography protocols may not be feasible for all participants due to tolerability or signal instability, potentially reducing completeness of some secondary measures. As an observational study, causal inference is limited, and confounding will require careful adjustment despite prespecified statistical analyses.

## Introduction

Neuropathic pain is a type of pain that occurs due to a lesion or disease of the somatosensory system [1]. It is linked to poorer quality of life, increased emotional distress, and higher healthcare utilisation compared to other forms of chronic non-neuropathic pain, such as arthritic pain [2]. Peripheral neuropathy, which is injury to peripheral nerve fibres, is the most common cause. Nearly 10% of the population suffer from peripheral neuropathy [3], with diabetes mellitus the leading cause [4]. In the UK, diabetes prevalence is projected to rise from 6.3% in 2021 to 7.5% by 2045. Diabetic distal symmetrical polyneuropathy affects 30–50% of individuals with diabetes and about half will suffer from chronic neuropathic pain [4]. Rarely, neuropathic pain can result from genetic [5] or immune-mediated changes [6] in ion channel function that leads to peripheral nerve hyperexcitability.

Chronic neuropathic pain remains underdiagnosed, and current treatments offer limited efficacy and tolerability [1]. Experts increasingly advocate mechanism-based treatment approaches [7], yet clinicians still lack robust information to match specific therapies to individual patients [8]. A major reason is the way we currently classify neuropathic pain. Clinicians often base diagnosis on aetiology, even though underlying mechanisms and clinical features can differ within the same aetiology and overlap across different aetiologies. Genetic, biochemical, environmental and psychosocial factors shape the complex pathophysiology of neuropathic pain [4,9–13]. To improve outcomes, we need classification schemes and treatment algorithms that target specific pathophysiological pathways.

Abnormal excitability and activity of peripheral nociceptor fibres appear to drive pain in a subset of people with chronic neuropathic pain [14,15]. Both human and animal studies link neuropathic pain to peripheral neuronal hyperexcitability, and human studies indicate that peripheral nociceptor input can maintain neuropathic pain. For example, topical or systemic lidocaine reduces spontaneous neuropathic pain and mechanical and thermal hypersensitivity in patients with peripheral nerve injury or distal symmetrical polyneuropathy [16]. Pre-clinical models implicate altered expression and gating of voltage-gated sodium channels (NaV) in injured nociceptive fibres as mechanisms for hyperexcitability [14]. Human genetic studies corroborate these findings. For example, rare gain- or loss-of-function mutations in nociceptor channels (e.g., TRPA1, NaV subtypes) cause extreme pain syndromes or insensitivity to pain [5,17]. While variants in the same genes are linked to more common neuropathies such as diabetes and small-fibre neuropathy [10,18]. Investigation of rare genetic variants provides important clues to the molecular mechanisms of neuropathic pain. Measuring nociceptive fibre activity directly can identify those whose pain is driven primarily by peripheral fibre hyperexcitability, thereby uncovering key pathophysiological processes and guiding targeted therapy.

However, human studies have not fully characterised which nociceptor populations are abnormal, how neuronal abnormalities relate to clinical phenotype, or the precise molecular changes involved. Many attempts have stratified patients by clinical and sensory profiles, for example via quantitative sensory testing. [19]. Quantitative sensory testing is a standardised and validated psychophysical tool for the systematic assessment and quantification of an individual's pain perception by applying controlled stimuli to areas of pain [20]. This helps in the objective evaluation of pain conditions and responses. But, these sensory profiles have not been tightly linked to direct measures of nociceptor activity. Surrogate neurophysiological measures such as, large-fibre axonal excitability, do not reliably differentiate painful from painless neuropathies [21]. Direct recordings from nociceptors are therefore essential.

Microneurography is the only neurophysiological tool that records neuronal activity directly from nerve fibres in awake humans [22]. It allows assessment of thin myelinated Aδ and unmyelinated C-fibres (largely composed of nociceptive fibres), which cannot be assessed by conventional nerve conduction studies nor axonal excitability [23]. It is thus ideally suited to investigate peripheral mechanisms underlying neuropathic pain [24,25]. Microneurography studies have shown nociceptor hyperexcitability in pathological states including diabetes and individuals with NaV channel mutations [26–37]. For example, certain NaV1.7 pathogenic variants increase spontaneous activity in previously “silent” nociceptors, whereas other NaV1.8 variants reduce spontaneous firing, demonstrating variant-specific effects across nociceptor subtypes [38–40]. This opens the possibility of personalised, mechanism-targeted treatments.

Nevertheless, it remains unclear how nociceptor populations are affected across different painful neuropathies, how neuronal excitability correlates with clinical and sensory phenotypes, and which molecular mechanisms underlie these changes. This study will investigate these questions.

## Methods

We will perform an observational controlled study at the University of Oxford and report findings according to STROBE [41]. Participant recruitment began in February 2022, and both recruitment and data collection are scheduled for completion in September 2029. Results will follow in the subsequent year.

### Study participants

Healthy: adults (≥16 years) without risk factors or clinical features of peripheral neuropathy.

Patient participants: adults (≥16 years) with risk factors for neuropathy (for example diabetes), symptoms suggestive of neuropathy, or a diagnosis of diabetic or non-diabetic peripheral neuropathy, including small-fibre neuropathy, established by clinical assessment and investigations. Also included are patients (or their first-degree relatives) with clinical features or a confirmed diagnosis of paroxysmal extreme pain disorder, familial episodic pain syndrome, erythromelalgia, or congenital insensitivity to pain.

Exclusion criteria: pregnancy; insufficient English or impaired capacity preventing informed consent or completion of questionnaires; severe psychiatric illness; significant pain from other causes that would confound assessment; central nervous system lesions likely to complicate somatosensory testing; or any other reason the investigator deems the participant unsuitable.

### Study participant recruitment

Participants will be recruited from multiple sources to capture broad neuropathy and pain severity spectra: specialist neuropathy and clinical neurophysiology clinics, diabetic podiatry clinics, primary care networks such as the Thames Valley Primary Care Research Partnership, advertisements, and previous study cohorts where consent permits recontact.

### Ethical approval and consent to participate

Ethical approval was obtained from the South Central – Oxford C Research Ethics Committee (18/SC/0263). All participants will provide informed written or electronic consent in accordance with the Declaration of Helsinki. Adverse events during microneurography or other study procedures will be recorded. Protocol amendments will be approved by the ethics committee, registered and communicated to collaborators.

### Tools for phenotyping and study procedures

Patient participants will undergo a deep clinical phenotyping [42] and then divided into groups based on aetiology, neuropathy, and neuropathic pain grading [10].

The tools for phenotyping and study procedures closely align with the DOLORisk protocol, which incorporates questionnaires, clinical assessments, and specialised investigations.[42]

### Questionnaires

#### Demographic and lifestyle information.

The study records demographic information, including age, gender, weight, height, and smoking and alcohol consumption.

#### *Characterisation of pain – pain location, intensity, quality, analgesics*.

We assess the presence and duration of pain, including dysaesthesia. Participants are asked to indicate where they feel pain on their body using a list of body sites and a body map. They identify all areas where they have experienced pain over the past three months and mark the location that bothers them the most.

Pain intensity is measured using different scoring systems such as the Chronic Pain Grade [43] over the past three months and the Brief Pain Inventory [44]. An additional item asks about the average pain experienced over the past seven days. Pain descriptors are characterised by the DN4 (*Douleur Neuropathique en 4 questions*) [45], and the Neuropathic Pain Symptom Inventory [46]. These questionnaires use an 11-point numerical scale to quantify severity of pain and symptoms.

The Michigan Neuropathy Screening Instrument is a screening tool for diabetic neuropathy and asks participants about symptoms related to diabetic neuropathy, such as pain, numbness, tingling, and loss of sensation in the feet and legs [47].

We record details of pain medication (e.g., paracetamol, gabapentin), dose, analgesic relief, and adherence to medication.

#### Pain interference, quality of life and psychological variables.

The Patient-Reported Outcomes Measurement Information System (PROMIS) questionnaires are used to assess several psychological and psychosocial variables [48]. These include depression, anxiety, sleep disturbance, fatigue and pain interference. Other psychological questionnaires include Ten-Item Personality Inventory [49], and assessments of Pain Related Worrying [50] and State Optimism Measure [51].

The EQ-5D [52] measures quality of life with a visual analogue scale and five items evaluating the impact of pain on the ability of the participant to perform everyday tasks.

### Clinical neurological assessment

A comprehensive structured upper and lower limb neurological examination is performed to detect clinical signs of a peripheral neuropathy. The examination includes assessment of muscle bulk, tone, motor power, deep-tendon reflexes (using a Queen square tendon hammer). The sensory examination assesses vibration perception (using a 128 Hz tuning fork), pinprick sensation (using ‘Neurotip’), temperature (using Somedic RollTemp, Somedic AB, Sweden), light touch (using 10g monofilament) and, joint position sense.

The clinical findings for a length-dependent neuropathy are quantified with the Toronto Clinical Scoring System (TCSS) [53].

### Specialised investigations

#### Microneurography.

Microneurography records single-unit activity from peripheral sensory nerves in awake participants by inserting a microelectrode into a peripheral nerve [54]. We typically use the superficial peroneal nerve on the dorsum of the foot because it is superficial, purely sensory, anatomically relevant to distal symmetrical polyneuropathy, and generally well tolerated. It is also the most distal nerve available, which provides an advantage for length-dependent (‘dying-back’) neuropathies that show a distal-to-proximal gradient. In older participants (>70 years) or those with severe neuropathy where the superficial peroneal nerve is denervated or inaccessible, alternative sites (superficial radial nerve or more proximal peroneal sites) will be used.

C-fibres are characterised by the phenomenon of activity-dependent slowing of conduction velocity in response to repetitive transcutaneous electrical stimuli, and natural stimulation in a series of experiments [24].

The core protocol will consist of:

Identification of C-fibres at 0.25 Hz stimulation. After identification, stimulation is paused for three minutes to allow baseline recovery, followed by six minutes at 0.25 Hz. The rate is then increased to 2 Hz for three minutes and followed by a further six minutes at 0.25 Hz. The pause and 0.25 Hz blocks may be adjusted for participant tolerability, but the 2 Hz block will not be shortened.

Where possible this will be followed by the extended protocol.

1. Stimulation at 0.125 Hz, 0.25 Hz, 0.5 Hz and 1 Hz for at least two minutes at each frequency.
2. Paired-pulse testing to assess recovery cycles by delivering a test stimulus at progressively shorter intervals after a conditioning stimulus.
3. Mechanical and thermal natural stimulation of the receptive field using von Frey hairs and thermal probes (40°C, 45°C, 48°C and 20°C) to determine transduction thresholds and sensitisation to mechanical and thermal stimulation.

We aim to record from up to three independent receptive fields per participant, but a single stable recording suffices for inclusion. All C-fibres will undergo the core protocol; the extended protocol is optional and recorded whenever feasible.

C-fibre subtypes are classified according to their response to an increase in electrical stimulation frequency to 2 Hz. This response, known as activity-dependent slowing of conduction velocity, is a characteristic feature of C-fibres in which conduction latency increases (i.e., slows) following repetitive stimulation. Using this method, five distinct populations of C-fibres can be identified: type 1A (“mechano-sensitive”) nociceptors, type 1B (“mechano-insensitive”) nociceptors, cold thermoreceptors, low-threshold mechanoreceptors, and cutaneous sympathetic efferent fibres [24,25].

There are two main reasons for distinguishing between a core and an extended recording protocol. The first relates to participant tolerability. Some individuals, particularly older participants, find prolonged recordings difficult due to musculoskeletal discomfort, difficulty keeping the limb still, challenges in following instructions, or urinary urgency. To minimise discomfort, participants are seated in a supportive chair with pillows to cushion pressure points, and are offered comfort breaks at the start of the recording. The second reason concerns recording stability. C-fibre signals may be lost during longer sessions because of small movements of the electrode or limb, or loss of contact during natural stimulation. As a result, extended recordings cannot always be completed. To prevent ascertainment bias, data from the extended protocol will therefore be treated as secondary or exploratory outcomes.

Microneurography measurements will include measures of:

Axonal properties

i. Baseline conduction velocity.
ii. Degree of activity-dependant slowing to several stimulation frequencies (e.g., 0.25 Hz to 2 Hz)
iii. Action potential morphology analysis of the extracellular recorded action potentials
iv. Threshold responses to thermal and mechanical stimulation

Nociceptor excitability

i. Spontaneous activity
ii. Double or multiple spikes
iii. Thermal and mechanical sensitisation defined as prolonged ongoing discharges outlasting stimulus duration or lowered thresholds in response to thermal and mechanical stimulation.

Axonal excitability (across all C-fibre types)

i. The velocity recovery cycle refers to the interval-dependent change in C-fibre conduction velocity after an action potential. It is used to measure refractoriness and excitability. It progresses through several distinct phases that depend on the time interval between the first (conditioning) action potential and the second (test) action potential. These phases include the refractory period, the supernormal period, and the late subnormal period.

#### Nerve conduction studies.

Nerve conduction studies are performed to confirm the presence of a distal symmetrical polyneuropathy, following the recommendations of the American Academy of Neurology and the American Association of Electrodiagnostic Medicine [55]. The initial assessment includes sural sensory and peroneal motor nerve conduction studies in one lower limb. If both results are normal, no further testing is required to exclude large-fibre distal symmetrical polyneuropathy.

If either study is abnormal, additional tests are undertaken as appropriate. These may include assessments of the ipsilateral tibial motor nerve; the contralateral sural sensory, peroneal motor, or tibial motor nerves; or the median sensory and ulnar sensory and motor nerves in one upper limb.

Electrodiagnostic confirmation of a length-dependent distal symmetrical polyneuropathy is defined as an abnormality in any attribute of nerve conduction in at least two separate nerves, one of which must be the sural nerve. Variables such as skin temperature, age, and height are taken into account when interpreting results.

#### Axonal excitability.

In selected cases, axonal excitability will be assessed using an electrophysiological technique known as threshold tracking [23]. Nerve excitability reflects the biophysical properties of myelinated axons and their membrane potential. These measurements complement conventional nerve conduction studies, which primarily assess action potential amplitude and latency but provide limited information about underlying membrane dynamics. In contrast, threshold tracking is sensitive to changes in membrane potential at the site of stimulation.

Key parameters include refractoriness, supernormality, the strength–duration time constant, and threshold electrotonus. Recordings will be obtained from the motor and sensory divisions of the median nerve in accordance with published recommendations [56]. This approach also enables modelling of ion channel function.

#### Quantitative sensory testing.

Quantitative sensory testing is a psychophysical technique used to assess sensory perception in response to defined stimuli. It provides a quantitative measure of sensory function and generates a sensory profile that captures both loss and gain of function across multiple sensory modalities. Quantitative sensory testing will be performed according to a modified version [42] of the German Research Network on Neuropathic Pain (DFNS) protocol [20,57]. In this study population, most participants are expected to experience bilateral symptoms; therefore, testing will be performed unilaterally on the dorsum of the foot and/or hand.

Quantitative sensory testing data will be entered into the Equista software package (version 1.2.2., CASQUAR GmbH), developed by the German Research Network on Neuropathic Pain. Equista converts raw data into z-scores, normalising results for age, sex, and the body region tested [58]. A z-score of zero represents the population mean, while scores greater than +2 or less than –2 standard deviations indicate gain or loss of function, respectively.

Participants will be stratified into somatosensory phenotype groups based on their quantitative sensory testing results. Two complementary approaches will be used:

1. Cluster analysis – An unbiased algorithm identifies three distinct phenotypic groups: sensory loss, mechanical hyperalgesia, and thermal hyperalgesia. Individuals will be assigned deterministically to one of these groups [13,19].
2. Irritable versus non-irritable phenotype – Participants will be classified as having an irritable phenotype when detection thresholds are normal but there is evidence of sensory gain (mechanical or thermal hyperalgesia), or a non-irritable phenotype when there is either sensory loss or no evidence of sensory gain [59].

#### Skin biopsy.

Intra-epidermal nerve fibre density is a validated measure for assessing small-fibre pathology. Skin biopsy samples will be obtained in accordance with the published guidelines of the European Federation of Neurological Societies/Peripheral Nerve Society on the use of skin biopsy in the diagnosis of peripheral neuropathies [60]. The biopsy will be taken at the end of the clinical assessment, from a site located 10 cm proximal to the lateral malleolus, once all other relevant investigations have been completed.

Participants will not undergo a skin biopsy if they decline the procedure, are receiving warfarin or other anticoagulant therapy, or have contraindications such as local skin infection or ulceration.

#### Genotyping.

This study uses DNA and RNA sequencing to investigate the genetic and molecular mechanisms that drive changes in C-fibre axonal properties and excitability. We will collect blood samples to extract DNA and analyse genetic variants that may influence C-fibre axonal properties and nociceptor excitability, focusing on genes involved in ion channel function and peripheral nerve signalling. We will extract RNA from blood and skin samples to characterise gene expression using high-throughput RNA sequencing, enabling us to identify transcriptional changes that modify C-fibre excitability and sensory transduction. Sequencing data will be processed through validated bioinformatic pipelines, with key findings verified using quantitative methods such as RT-PCR.

Together, these approaches will reveal how genetic and molecular factors shape C-fibre function by identifying variants and expression changes linked to altered axonal behaviour and nociceptor properties.

### Data analysis

#### Patient stratification.

Patient study participant’s neuropathy [61] and pain [1] is graded according to published guidelines. The diagnosis of distal symmetrical polyneuropathy (DSP) is based on a detailed neurological assessment in combination with tests.

**Table:** 

No DSP	No abnormalities on clinical assessment or tests
Subclinical DSP	Abnormal tests but no signs or symptoms
Possible DSP	Symptoms: decreased sensation, positive neuropathic sensory symptoms in the toes, feet, or legs, e.g., numbness, burning; or Signs: symmetric decrease of distal sensation or unequivocally decreased or absent ankle reflexes
Probable DSP	Two or more: neuropathic symptoms, decreased distal sensation, or unequivocally decreased or absent ankle reflexes
Confirmed DSP	Abnormal nerve conduction tests or a measure of small-fibre dysfunction (intraepidermal nerve fibre density or thermal thresholds), and abnormal clinical assessment (a symptom or sign)

Participants are then graded according to the presence or absence of neuropathic pain. Neuropathic pain classification is based on whether the pain present is neuropathic in nature and follows a distinct neuroanatomically plausible distribution.

Participants included in analysis will be divided into groups based on aetiology, neuropathy, and neuropathic pain grading [10].

i. **Diabetic participants** are divided into those with (possible/probable/confirmed) and without a distal symmetrical polyneuropathy
ii. **Diabetic participants with a distal symmetrical polyneuropathy** are further divided into those with and without neuropathic pain.
iii. **Non-diabetic participants** with distal symmetrical polyneuropathy are divided into those with and without neuropathic pain. This will include participants with small-fibre neuropathy, i.e., those participants with injury confined to the small fibres (Aδ and C-fibres.)
iv. **Extreme pain phenotypes** include:

**Erythromelalgia** and **paroxysmal extreme pain disorder** – episodic or persistent pain and erythema of the extremities or trunk that is exacerbated by warming and relieved by cooling;

**Familial episodic pain syndrome** – severe episodic pain localised to the trunk and limbs with no structural cause;

**Insensitivity to pain** – inability to feel nociceptive stimuli

#### Study hypotheses.

The primary hypothesis is that participants with neuropathic pain will exhibit altered axonal properties and excitability compared to those without neuropathic pain.

Secondary hypotheses we will test are:

1) Somatosensory subgroups will match nociceptor properties. The planned outcomes are comparisons between neurophysiological and sensory phenotype variables, for example, mechanical hyperalgesia sensory profile will show lowered neurophysiological threshold to mechanical stimulation.
2) Genetic variants will confer a unique nociceptive axonal profile and excitability signature. The planned outcome is the comparison of nociceptor fibre excitability and axonal properties between those participants with and without genetic variants.

#### Statistical plan.

Analyses will address the relationships between neuropathy, neuropathic pain, quantitative sensory phenotype and microneurography measures of nociceptor excitability and axonal properties. Analyses will be prespecified, scripted for reproducibility and conducted using appropriate statistical software.

Continuous variables will be summarised using mean (standard deviation) or median (interquartile range) depending on distribution; categorical variables will be summarised using counts and percentages. Point estimators will be reported alongside their respective 95% confidence interval. All hypothesis tests will be two-tailed. Where multiple comparisons are performed, p-values will be adjusted using methods appropriate to the comparison (for example Bonferroni or false discovery rate), and this will be reported alongside unadjusted p-values.

The primary hypothesis is that participants with neuropathic pain will exhibit altered axonal properties and excitability compared to those without neuropathic pain. The primary outcome will therefore be a comparison of microneurography measures of axonal excitability and axonal properties between participants with and without neuropathic pain.

#### Primary outcome comparisons.

Comparison of mean values and distributions of axonal excitability and axonal property measures at participant level for individual nociceptive fibres (for example, spontaneous activity, multiple spikes, mechanical and thermal sensitisation, conduction velocity, and activity-dependent slowing).

#### Planned primary analysis.

Between-group comparisons (neuropathic pain vs no neuropathic pain) will be conducted using appropriate non-parametric tests (Mann–Whitney U test) where data are not normally distributed, or independent-samples t-tests where normality is satisfied.

Data from individual nociceptive fibres will be analysed with explicit consideration of clustering within participants. Where appropriate, fibre-level measurements will be summarised at the participant level (for example, by averaging) to provide an independent observation per participant for primary analyses. In addition, mixed-effects models will be used, with participant included as a random effect, to account for within-participant variability. Multiplicity arising from multiple outcomes will be addressed using appropriate multiple-comparison adjustments (for example, Bonferroni or false discovery rate correction).

This dual approach allows us to perform conservative primary analyses at the participant level by averaging, while also utilising more sophisticated modelling through a mixed-effects model that retains the detail of the individual fibre data while correcting for the dependence between fibres from the same participant. The mixed-effects model will bridge the participant (unit of inference) and individual fibre (unit of observation) without the statistical error of treating fibres from the same person as independent data points.

Multivariable logistic regression models will be used to examine the association between neuropathic pain status and microneurography-derived measures of axonal excitability and properties, adjusting for prespecified covariates (age, sex, diabetes status, neuropathy severity, and medication use).

Multicollinearity among predictors will be evaluated using variance inflation factors; when two predictors are highly correlated, the variable with greater variance or stronger biological relevance will be retained in the final model.

#### Secondary analyses.

Secondary hypotheses explore relationships between somatosensory phenotype (QST clusters), genetic variants and nociceptor excitability signatures.

1. Somatosensory subgroup correspondence

- Compare microneurography variables across QST-defined phenotypic groups (sensory loss; mechanical hyperalgesia; thermal hyperalgesia) using non-parametric ANOVA (Kruskal–Wallis) or one-way ANOVA as appropriate.

- If omnibus tests are significant, perform post-hoc comparisons with correction for multiple testing.

2. Genetic variant effects

- Compare microneurography measures between participants who carry rare variants in ion channel genes (NaV, TRPA1) and those who do not, stratified by aetiology (diabetic versus non-diabetic).

- Use regression models to estimate the effect of genetic variants on fibre excitability, adjusting for confounders. If sample sizes for specific variants are small, present descriptive statistics and cautionary interpretation.

3. Microneurography association analyses

- Spearman rank-order correlations will evaluate associations between microneurography measures and clinical metrics: spontaneous pain intensity, evoked pain intensity, DN4/NPSI descriptor scores, and quality-of-life measures.

- A binomial regression model will examine the association between microneurography variables and membership of somatosensory subgroups.

Dimensionality reduction and clustering

Given the high dimensionality of clinical, genetic, microneurographic and biochemical data, we will use dimensionality reduction and unsupervised clustering to identify latent patient subgroups.

- Apply principal component analysis to standardised variables across the domains of interest (genetics, demographics, microneurography, biochemistry). Retain components that explain an adequate cumulative variance (e.g., eigenvalues >1).

- Apply k-means, centroid and hierarchical clustering on retained principal components. Determine optimal cluster number using multiple metrics (gap statistic, silhouette width, elbow method).

- After cluster assignment, compare original variables across clusters using omnibus tests with post-hoc comparisons to characterise cluster profiles.

#### Sample size justification.

Sample size calculations are based on power to identify differences in QST cluster frequencies and to support clustering and multivariate analyses.

- Using pilot quantitative sensory testing data from DOLORisk, expected cluster frequencies for diabetic distal symmetrical polyneuropathy are 53.9% (sensory loss), 25.0% (mechanical hyperalgesia) and 15.8% (thermal hyperalgesia). For non-diabetic neuropathies the corresponding frequencies are 49.7%, 21.2% and 14.5%.

- For moderate effects (Cohen’s d ~ 0.5) in the quantitative sensory testing cluster analyses, a total of 130 diabetic and 148 non-diabetic participants provides approximately 80% power at α = 0.05.

- For k-means and hierarchical clustering applied after principal component analysis, a conservative estimate requiring 2^8 = 256 participants was used to ensure adequate representation across variable permutations.

- A multivariate regression model with age, sex, diabetes status, neuropathy severity, and medication use will require 8 degrees of freedom to represent all its variables (assuming a categorical variable coding for 4 categories of medication). Allowing for at least 15 event per variable (EPV) we will require a sample size of at least 120 participants for model development. As described above, variable selection and filtering based on multicollinearity will likely increase the EPV.

- Taking these calculations together (and accounting for anticipated success rates in microneurography recordings: minimum 60% for diabetic participants and 80% for non-diabetic participants), the recruitment target is 272 participants (124 diabetic and 148 non-diabetic).

#### Risk of bias and mitigation.

Potential sources of bias include misclassification of fibre type, sampling bias, outcome reporting bias and confounding.

Fibre classification bias will be minimised using pre-specified, reproducible criteria based on activity-dependent slowing of conduction velocity. Sampling bias will be reduced by recording from multiple receptive fields per participant and using mixed-effects models to account for within-participant variability. Confounders will be adjusted for in regression models. The protocol and statistical analysis plan will be pre-specified and published to minimise outcome reporting bias.

### Data management

All study data will be recorded on case report forms and managed using REDCap. Identifiable data will be stored separately and securely, accessible only to authorised team members. Electronic data will be stored on secure, password-protected servers behind institutional firewalls; paper records will be kept in locked cabinets in secure areas. Data entries will be randomly checked for accuracy by an independent researcher (up to 30%).

As this is an observational study there is no external monitoring committee but we will internally monitor and regularly audit data collection and delivery of interventions. All research data and records will be stored in accordance with data protection and University of Oxford policies.

## References

[1] Finnerup NB, Haroutounian S, Kamerman P, Baron R, Bennett DLH, Bouhassira D, et al. Neuropathic pain: an updated grading system for research and clinical practice. Pain. 2016;157(8):1599–606. doi: 10.1097/j.pain.0000000000000492 27115670 PMC4949003

[2] Attal N, Lanteri-Minet M, Laurent B, Fermanian J, Bouhassira D. The specific disease burden of neuropathic pain: results of a French nationwide survey. Pain. 2011;152(12):2836–43. doi: 10.1016/j.pain.2011.09.014 22019149

[3] Martyn CN, Hughes RA. Epidemiology of peripheral neuropathy. J Neurol Neurosurg Psychiatry. 1997;62(4):310–8. doi: 10.1136/jnnp.62.4.310 9120441 PMC1074084

[4] Feldman EL, Nave K-A, Jensen TS, Bennett DLH. New Horizons in Diabetic Neuropathy: Mechanisms, Bioenergetics, and Pain. Neuron. 2017;93(6):1296–313. doi: 10.1016/j.neuron.2017.02.005 28334605 PMC5400015

[5] Bennett DLH, Woods CG. Painful and painless channelopathies. Lancet Neurol. 2014;13(6):587–99. doi: 10.1016/S1474-4422(14)70024-9 24813307

[6] Dawes JM, Weir GA, Middleton SJ, Patel R, Chisholm KI, Pettingill P, et al. Immune or Genetic-Mediated Disruption of CASPR2 Causes Pain Hypersensitivity Due to Enhanced Primary Afferent Excitability. Neuron. 2018;97(4):806-822.e10. doi: 10.1016/j.neuron.2018.01.033 29429934 PMC6011627

[7] Labau JIR, Estacion M, Tanaka BS, de Greef BTA, Hoeijmakers JGJ, Geerts M. Differential effect of lacosamide on Nav1.7 variants from responsive and non-responsive patients with small fibre neuropathy. Brain. 2020;143(3):771–82. doi: 10.1093/brain/awaa016 32011655 PMC7089662

[8] Baron R, Dickenson AH, Calvo M, Dib-Hajj SD, Bennett DL. Maximizing treatment efficacy through patient stratification in neuropathic pain trials. Nat Rev Neurol. 2023;19(1):53–64. doi: 10.1038/s41582-022-00741-7 36400867

[9] Baskozos G, Themistocleous AC, Hebert HL, Pascal MMV, John J, Callaghan BC, et al. Classification of painful or painless diabetic peripheral neuropathy and identification of the most powerful predictors using machine learning models in large cross-sectional cohorts. BMC Med Inform Decis Mak. 2022;22(1):144. doi: 10.1186/s12911-022-01890-x 35644620 PMC9150351

[10] Themistocleous AC, Baskozos G, Blesneac I, Comini M, Megy K, Chong S, et al. Investigating genotype-phenotype relationship of extreme neuropathic pain disorders in a UK national cohort. Brain Commun. 2023;5(2):fcad037. doi: 10.1093/braincomms/fcad037 36895957 PMC9991512

[11] Themistocleous AC, Ramirez JD, Shillo PR, Lees JG, Selvarajah D, Orengo C, et al. The Pain in Neuropathy Study (PiNS): a cross-sectional observational study determining the somatosensory phenotype of painful and painless diabetic neuropathy. Pain. 2016;157(5):1132–45. doi: 10.1097/j.pain.0000000000000491 27088890 PMC4834814

[12] Freeman R, Baron R, Bouhassira D, Cabrera J, Emir B. Sensory profiles of patients with neuropathic pain based on the neuropathic pain symptoms and signs. Pain. 2014;155(2):367–76. doi: 10.1016/j.pain.2013.10.023 24472518

[13] Vollert J, Maier C, Attal N, Bennett DLH, Bouhassira D, Enax-Krumova EK, et al. Stratifying patients with peripheral neuropathic pain based on sensory profiles: algorithm and sample size recommendations. Pain. 2017;158(8):1446–55. doi: 10.1097/j.pain.0000000000000935 28595241 PMC5515640

[14] Roza C, Bernal L. Electrophysiological characterization of ectopic spontaneous discharge in axotomized and intact fibers upon nerve transection: a role in spontaneous pain?. Pflugers Arch. 2022;474(4):387–96. doi: 10.1007/s00424-021-02655-7 35088129

[15] Finnerup NB, Kuner R, Jensen TS. Neuropathic Pain: From Mechanisms to Treatment. Physiol Rev. 2021;101(1):259–301. doi: 10.1152/physrev.00045.2019 32584191

[16] Haroutounian S, Nikolajsen L, Bendtsen TF, Finnerup NB, Kristensen AD, Hasselstrøm JB, et al. Primary afferent input critical for maintaining spontaneous pain in peripheral neuropathy. Pain. 2014;155(7):1272–9. doi: 10.1016/j.pain.2014.03.022 24704366

[17] Bennett DL, Clark AJ, Huang J, Waxman SG, Dib-Hajj SD. The Role of Voltage-Gated Sodium Channels in Pain Signaling. Physiol Rev. 2019;99(2):1079–151. doi: 10.1152/physrev.00052.2017 30672368

[18] Blesneac I, Themistocleous AC, Fratter C, Conrad LJ, Ramirez JD, Cox JJ, et al. Rare NaV1.7 variants associated with painful diabetic peripheral neuropathy. Pain. 2018;159(3):469–80. doi: 10.1097/j.pain.0000000000001116 29176367 PMC5828379

[19] Baron R, Maier C, Attal N, Binder A, Bouhassira D, Cruccu G, et al. Peripheral neuropathic pain: a mechanism-related organizing principle based on sensory profiles. Pain. 2017;158(2):261–72. doi: 10.1097/j.pain.0000000000000753 27893485 PMC5266425

[20] Rolke R, Baron R, Maier C, Tölle TR, Treede - D R, Beyer A, et al. Quantitative sensory testing in the German Research Network on Neuropathic Pain (DFNS): standardized protocol and reference values. Pain. 2006;123(3):231–43. doi: 10.1016/j.pain.2006.01.041 16697110

[21] Themistocleous AC, Kristensen AG, Sola R, Gylfadottir SS, Bennedsgaard K, Itani M, et al. Axonal Excitability Does Not Differ between Painful and Painless Diabetic or Chemotherapy-Induced Distal Symmetrical Polyneuropathy in a Multicenter Observational Study. Ann Neurol. 2022;91(4):506–20. doi: 10.1002/ana.26319 35150149 PMC9313833

[22] Ackerley R, Watkins RH. Microneurography as a tool to study the function of individual C-fiber afferents in humans: responses from nociceptors, thermoreceptors, and mechanoreceptors. J Neurophysiol. 2018;120(6):2834–46. doi: 10.1152/jn.00109.2018 30256737

[23] Kiernan MC, Bostock H, Park SB, Kaji R, Krarup C, Krishnan AV, et al. Measurement of axonal excitability: Consensus guidelines. Clin Neurophysiol. 2020;131(1):308–23. doi: 10.1016/j.clinph.2019.07.023 31471200

[24] Serra J, Campero M, Ochoa J, Bostock H. Activity-dependent slowing of conduction differentiates functional subtypes of C fibres innervating human skin. J Physiol. 1999;515 (Pt 3) (Pt 3):799–811. doi: 10.1111/j.1469-7793.1999.799ab.x 10066906 PMC2269177

[25] Middleton SJ, Barry AM, Comini M, Li Y, Ray PR, Shiers S, et al. Studying human nociceptors: from fundamentals to clinic. Brain. 2021;144(5):1312–35. doi: 10.1093/brain/awab048 34128530 PMC8219361

[26] Serra J, Bostock H, Solà R, Aleu J, García E, Cokic B, et al. Microneurographic identification of spontaneous activity in C-nociceptors in neuropathic pain states in humans and rats. Pain. 2012;153(1):42–55. doi: 10.1016/j.pain.2011.08.015 21993185

[27] Kleggetveit IP, Namer B, Schmidt R, Helås T, Rückel M, Ørstavik K, et al. High spontaneous activity of C-nociceptors in painful polyneuropathy. Pain. 2012;153(10):2040–7. doi: 10.1016/j.pain.2012.05.017 22986070

[28] Serra J, Collado A, Solà R, Antonelli F, Torres X, Salgueiro M, et al. Hyperexcitable C nociceptors in fibromyalgia. Ann Neurol. 2014;75(2):196–208. doi: 10.1002/ana.24065 24243538

[29] Ørstavik K, Namer B, Schmidt R, Schmelz M, Hilliges M, Weidner C, et al. Abnormal function of C-fibers in patients with diabetic neuropathy. J Neurosci. 2006;26(44):11287–94. doi: 10.1523/JNEUROSCI.2659-06.2006 17079656 PMC6674548

[30] Schmidt R, Kleggetveit IP, Namer B, Helås T, Obreja O, Schmelz M, et al. Double spikes to single electrical stimulation correlates to spontaneous activity of nociceptors in painful neuropathy patients. Pain. 2012;153(2):391–8. doi: 10.1016/j.pain.2011.10.041 22154219

[31] Campero M, Serra J, Marchettini P, Ochoa JL. Ectopic impulse generation and autoexcitation in single myelinated afferent fibers in patients with peripheral neuropathy and positive sensory symptoms. Muscle Nerve. 1998;21(12):1661–7. doi: 10.1002/(sici)1097-4598(199812)21:12<1661::aid-mus6>3.0.co;2-n 9843066

[32] Campero M, Serra J, Ochoa JL. C-polymodal nociceptors activated by noxious low temperature in human skin. J Physiol. 1996;497 (Pt 2)(Pt 2):565–72. doi: 10.1113/jphysiol.1996.sp021789 8961196 PMC1161005

[33] Bostock H, Campero M, Serra J, Ochoa JL. Temperature-dependent double spikes in C-nociceptors of neuropathic pain patients. Brain. 2005;128(Pt 9):2154–63. doi: 10.1093/brain/awh552 15947060

[34] Ochoa JL, Campero M, Serra J, Bostock H. Hyperexcitable polymodal and insensitive nociceptors in painful human neuropathy. Muscle Nerve. 2005;32(4):459–72. doi: 10.1002/mus.20367 15973653

[35] Serra J, Campero M, Bostock H, Ochoa J. Two types of C nociceptors in human skin and their behavior in areas of capsaicin-induced secondary hyperalgesia. J Neurophysiol. 2004;91(6):2770–81. doi: 10.1152/jn.00565.2003 14762154

[36] Orstavik K, Jørum E. Microneurographic findings of relevance to pain in patients with erythromelalgia and patients with diabetic neuropathy. Neurosci Lett. 2010;470(3):180–4. doi: 10.1016/j.neulet.2009.05.061 19481586

[37] Ribeiro A, Hadavi S, Gall N, Hadden RDM, Serra J. Microneurography Reveals Unmyelinated Small Nerve Fiber Dysfunction in Long COVID. Ann Neurol. 2026;99(2):356–68. doi: 10.1002/ana.78045 40977575

[38] Namer B, Ørstavik K, Schmidt R, Kleggetveit I-P, Weidner C, Mørk C, et al. Specific changes in conduction velocity recovery cycles of single nociceptors in a patient with erythromelalgia with the I848T gain-of-function mutation of Nav1.7. Pain. 2015;156(9):1637–46. doi: 10.1097/j.pain.0000000000000229 25993546

[39] Kist AM, Sagafos D, Rush AM, Neacsu C, Eberhardt E, Schmidt R, et al. SCN10A Mutation in a Patient with Erythromelalgia Enhances C-Fiber Activity Dependent Slowing. PLoS One. 2016;11(9):e0161789. doi: 10.1371/journal.pone.0161789 27598514 PMC5012686

[40] Kleggetveit IP, Schmidt R, Namer B, Salter H, Helås T, Schmelz M, et al. Pathological nociceptors in two patients with erythromelalgia-like symptoms and rare genetic Nav 1.9 variants. Brain Behav. 2016;6(10):e00528. doi: 10.1002/brb3.528 27781142 PMC5064340

[41] Ghaferi AA, Schwartz TA, Pawlik TM. STROBE Reporting Guidelines for Observational Studies. JAMA Surg. 2021;156(6):577–8. doi: 10.1001/jamasurg.2021.0528 33825815

[42] Pascal MMV, Themistocleous AC, Baron R, Binder A, Bouhassira D, Crombez G, et al. DOLORisk: study protocol for a multi-centre observational study to understand the risk factors and determinants of neuropathic pain. Wellcome Open Res. 2019;3:63. doi: 10.12688/wellcomeopenres.14576.2 30756091 PMC6364377

[43] Von Korff M, Ormel J, Keefe FJ, Dworkin SF. Grading the severity of chronic pain. Pain. 1992;50(2):133–49. doi: 10.1016/0304-3959(92)90154-4 1408309

[44] Cleeland CS, Ryan KM. Pain assessment: global use of the Brief Pain Inventory. Ann Acad Med Singap. 1994;23(2):129–38. 8080219

[45] Bouhassira D, Attal N, Alchaar H, Boureau F, Brochet B, Bruxelle J, et al. Comparison of pain syndromes associated with nervous or somatic lesions and development of a new neuropathic pain diagnostic questionnaire (DN4). Pain. 2005;114(1–2):29–36. doi: 10.1016/j.pain.2004.12.010 15733628

[46] Bouhassira D, Attal N, Fermanian J, Alchaar H, Gautron M, Masquelier E, et al. Development and validation of the Neuropathic Pain Symptom Inventory. Pain. 2004;108(3):248–57. doi: 10.1016/j.pain.2003.12.024 15030944

[47] Feldman EL, Stevens MJ, Thomas PK, Brown MB, Canal N, Greene DA. A practical two-step quantitative clinical and electrophysiological assessment for the diagnosis and staging of diabetic neuropathy. Diabetes Care. 1994;17(11):1281–9. doi: 10.2337/diacare.17.11.1281 7821168

[48] Cella D, Riley W, Stone A, Rothrock N, Reeve B, Yount S, et al. The patient-reported outcomes measurement information system (PROMIS) developed and tested its first wave of adult self-reported health outcome item banks: 2005-2008. J Clin Epidemiol. 2010;63(11):1179–94. doi: 10.1016/j.jclinepi.2010.04.011 20685078 PMC2965562

[49] Gosling SD, Rentfrow PJ, Swann WB Jr. A very brief measure of the big-five personality domains. J Research in Personality. 2003;37(6):504–28. doi: 10.1016/s0092-6566(03)00046-1

[50] Sullivan MJL, Bishop SR, Pivik J. The Pain Catastrophizing Scale: Development and validation. Psychological Assessment. 1995;7(4):524–32. doi: 10.1037/1040-3590.7.4.524

[51] Millstein RA, Chung W-J, Hoeppner BB, Boehm JK, Legler SR, Mastromauro CA, et al. Development of the State Optimism Measure. Gen Hosp Psychiatry. 2019;58:83–93. doi: 10.1016/j.genhosppsych.2019.04.002 31026732 PMC6501845

[52] Herdman M, Gudex C, Lloyd A, Janssen M, Kind P, Parkin D, et al. Development and preliminary testing of the new five-level version of EQ-5D (EQ-5D-5L). Qual Life Res. 2011;20(10):1727–36. doi: 10.1007/s11136-011-9903-x 21479777 PMC3220807

[53] Bril V, Perkins BA. Validation of the Toronto Clinical Scoring System for diabetic polyneuropathy. Diabetes Care. 2002;25(11):2048–52. doi: 10.2337/diacare.25.11.2048 12401755

[54] Ackerley R, Watkins RH. Microneurography as a tool to study the function of individual C-fiber afferents in humans: responses from nociceptors, thermoreceptors, and mechanoreceptors. J Neurophysiol. 2018;120(6):2834–46. doi: 10.1152/jn.00109.2018 30256737

[55] England JD, Gronseth GS, Franklin G, Miller RG, Asbury AK, Carter GT, et al. Distal symmetric polyneuropathy: a definition for clinical research: report of the American Academy of Neurology, the American Association of Electrodiagnostic Medicine, and the American Academy of Physical Medicine and Rehabilitation. Neurology. 2005;64(2):199–207. doi: 10.1212/01.WNL.0000149522.32823.EA 15668414

[56] Kiernan MC, Mogyoros I, Burke D. Changes in excitability and impulse transmission following prolonged repetitive activity in normal subjects and patients with a focal nerve lesion. Brain. 1996;119 (Pt 6):2029–37. doi: 10.1093/brain/119.6.2029 9010007

[57] Rolke R, Magerl W, Campbell KA, Schalber C, Caspari S, Birklein F, et al. Quantitative sensory testing: a comprehensive protocol for clinical trials. Eur J Pain. 2006;10(1):77–88. doi: 10.1016/j.ejpain.2005.02.003 16291301

[58] Magerl W, Krumova EK, Baron R, Tölle T, Treede R-D, Maier C. Reference data for quantitative sensory testing (QST): refined stratification for age and a novel method for statistical comparison of group data. Pain. 2010;151(3):598–605. doi: 10.1016/j.pain.2010.07.026 20965658

[59] Demant DT, Lund K, Vollert J, Maier C, Segerdahl M, Finnerup NB, et al. The effect of oxcarbazepine in peripheral neuropathic pain depends on pain phenotype: a randomised, double-blind, placebo-controlled phenotype-stratified study. Pain. 2014;155(11):2263–73. doi: 10.1016/j.pain.2014.08.014 25139589

[60] Lauria G, Hsieh ST, Johansson O, Kennedy WR, Leger JM, Mellgren SI, et al. European Federation of Neurological Societies/Peripheral Nerve Society Guideline on the use of skin biopsy in the diagnosis of small fiber neuropathy. Report of a joint task force of the European Federation of Neurological Societies and the Peripheral Nerve Society. Eur J Neurol. 2010;17(7):903–12, e44-9. doi: 10.1111/j.1468-1331.2010.03023.x 20642627

[61] Tesfaye S, Boulton AJM, Dyck PJ, Freeman R, Horowitz M, Kempler P, et al. Diabetic neuropathies: update on definitions, diagnostic criteria, estimation of severity, and treatments. Diabetes Care. 2010;33(10):2285–93. doi: 10.2337/dc10-1303 20876709 PMC2945176

