# Supplementary material for: Defining the link between peripheral neuronal activity and neuropathic pain: observational study protocol to investigate in vivo neurophysiological properties of nociceptors in patients with chronic neuropathic pain
Source: PLoS One. 2026 Jul 16;21(7):e0349407. doi: 10.1371/journal.pone.0349407 (PMC13375024; doi:10.1371/journal.pone.0349407)
Supplement: Attachment — Submitted filename: Response to Reviewers.docx [file pone.0349407.s002.docx]

|  |  |
| --- | --- |
| West Wing, Level 6, John Radcliffe Hospital, Oxford, OX3 9DU  **Web:** www.ndcn.ox.ac.uk \| **Tel**: +44(0)1865 857312 \| **Email**: andreas.themistocleous@ndcn.ox.ac.uk |  |

23^th^ April 2026

Dear Reviewers and editorial team

Thank you for the opportunity to submit revisions for our manuscript, *“Defining the link between peripheral neuronal activity and neuropathic pain: observational study protocol to investigate in vivo neurophysiological properties of nociceptors in patients with chronic neuropathic pain”*.

Our responses to the comments are highlighted in red:

Comment #1. Clarify the primary endpoint and the principal analytical unit more explicitly.

The manuscript states that the primary outcome will compare microneurography measures of axonal excitability and axonal properties between participants with and without neuropathic pain. However, the protocol then presents both participant-level and fibre-level primary outcome comparisons. Later, in the bias mitigation section, the authors state that mixed-effects models will be used to account for within-participant variability due to multiple receptive fields/fibres. These are all reasonable elements, but the current presentation leaves some ambiguity regarding what the true primary endpoint is, what the primary unit of inference will be, and how multiplicity and dependence between fibres from the same participant will be handled in confirmatory analyses. A clearer hierarchy of primary / secondary outcomes and analytical levels would improve methodological transparency.

We thank the reviewer for this thoughtful and constructive comment. We agree that clearer specification of the primary endpoint, the principal unit of inference, and the analytical hierarchy strengthens the methodological transparency of the protocol.

We clarify that the primary endpoint is the between-group difference in microneurography-derived measures of axonal excitability and axonal properties between participants with and without neuropathic pain, as originally intended. And that the primary unit of inference is at the participant level. Even when including individual fibre-level data in mixed models we specifically include the participant as the random effect, this way we use the fibre as the unit of observation and maintain the participant as the unit of inference across the study.

The text on page 18 now reads “Comparison of mean values and distributions of axonal excitability and axonal property measures at a participant level for individual nociceptive fibres (for example, spontaneous activity, multiple spikes, mechanical and thermal sensitisation, conduction velocity, and activity-dependent slowing).”

On page 19, we have included the following text to clarify how multiplicity and dependence between fibres from the same participant will be handled in confirmatory analyses:

“Data from individual nociceptive fibres will be analysed with explicit consideration of clustering within participants. Where appropriate, fibre-level measurements will be summarised at the participant level (for example, by averaging) to provide an independent observation per participant for primary analyses. In addition, mixed-effects models will be used, with participant included as a random effect, to account for within-participant variability. Multiplicity arising from multiple outcomes will be addressed using appropriate multiple-comparison adjustments (for example, Bonferroni or false discovery rate correction).

This dual approach allows us to perform conservative primary analyses at the participant level by averaging, while also utilising more sophisticated modelling through a mixed-effects model that retains the detail of the individual fibre data while correcting for the dependence between fibres from the same participant. The mixed-effects model will bridge the participant (unit of inference) and individual fibre (unit of observation) without the statistical error of treating fibres from the same person as independent data points.”

Comment #2. The manuscript would benefit from a final round of language editing. There are a few noticeable typographical or grammatical issues in the current text, which do not undermine the science but do affect presentation. For example: “due a lesion”, “Neary 10%”, “a 11-point numerical scale in to quantify”, “activity dependant”, “Ethical approved”, and a broken sentence: “sensitisation.to mechanical and thermal stimulation”

Thank you for highlighting these errors. We completed the necessary edits.

Sincerely,

Dr Andreas Themistocleous

MRC Clinician Scientist and consultant in clinical neurophysiology

On behalf of all co-authors
