# Supplementary material for: Defining the link between peripheral neuronal activity and neuropathic pain: observational study protocol to investigate in vivo neurophysiological properties of nociceptors in patients with chronic neuropathic pain
Source: PLoS One. 2026 Jul 16;21(7):e0349407. doi: 10.1371/journal.pone.0349407 (PMC13375024; doi:10.1371/journal.pone.0349407)
Supplement: Peer review history

# Peer review: Decision Letter 0

**DOI:** 10.1371/journal.pone.0349407.r001

**Authors:** Armando Almeida

31 Mar 2026

PONE-D-26-06203

Defining the link between peripheral neuronal activity and neuropathic pain: observational study protocol to investigate in vivo neurophysiological properties of nociceptors in patients with chronic neuropathic pain

PLOS One

Dear Dr. Themistocleous,

Thank you for submitting your manuscript to PLOS ONE. After careful consideration, we feel that it has merit but does not fully meet PLOS ONE’s publication criteria as it currently stands. Therefore, we invite you to submit a revised version of the manuscript that addresses the points raised during the review process.

Please submit your revised manuscript by May 15 2026 11:59PM. If you will need more time than this to complete your revisions, please reply to this message or contact the journal office at plosone@plos.org. When you're ready to submit your revision, log on to https://www.editorialmanager.com/pone/ and select the 'Submissions Needing Revision' folder to locate your manuscript file.

Please include the following items when submitting your revised manuscript:

- A letter that responds to each point raised by the academic editor and reviewer(s). You should upload this letter as a separate file labeled 'Response to Reviewers'.
- A marked-up copy of your manuscript that highlights changes made to the original version. You should upload this as a separate file labeled 'Revised Manuscript with Track Changes'.
- An unmarked version of your revised paper without tracked changes. You should upload this as a separate file labeled 'Manuscript'.

If you would like to make changes to your financial disclosure, please include your updated statement in your cover letter. Guidelines for resubmitting your figure files are available below the reviewer comments at the end of this letter.

If applicable, we recommend that you deposit your laboratory protocols in protocols.io to enhance the reproducibility of your results. Protocols.io assigns your protocol its own identifier (DOI) so that it can be cited independently in the future. For instructions see: https://journals.plos.org/plosone/s/submission-guidelines#loc-laboratory-protocols. Additionally, PLOS ONE offers an option for publishing peer-reviewed Lab Protocol articles, which describe protocols hosted on protocols.io. Read more information on sharing protocols at https://plos.org/protocols?utm_medium=editorial-email&utm_source=authorletters&utm_campaign=protocols.

As the corresponding author, your ORCID iD is verified in the submission system and will appear in the published article. PLOS supports the use of ORCID, and we encourage all coauthors to register for an ORCID iD and use it as well. Please encourage your coauthors to verify their ORCID iD within the submission system before final acceptance, as unverified ORCID iDs will not appear in the published article. *Only*  the individual author can complete the verification step; PLOS staff *cannot*  verify ORCID iDs on behalf of authors.

We look forward to receiving your revised manuscript.

Kind regards,

Armando Almeida

Academic Editor

PLOS One

Journal requirements:

When submitting your revision, we need you to address these additional requirements.

1. Please ensure that your manuscript meets PLOS ONE's style requirements, including those for file naming. The PLOS ONE style templates can be found at

https://journals.plos.org/plosone/s/file?id=wjVg/PLOSOne_formatting_sample_main_body.pdf and

https://journals.plos.org/plosone/s/file?id=ba62/PLOSOne_formatting_sample_title_authors_affiliations.pdf

2. Please upload a copy of Figure 2, to which you refer in your text on page 11. If the figure is no longer to be included as part of the submission please remove all reference to it within the text.

3. Your ethics statement should only appear in the Methods section of your manuscript. If your ethics statement is written in any section besides the Methods, please delete it from any other section.

If the reviewer comments include a recommendation to cite specific previously published works, please review and evaluate these publications to determine whether they are relevant and should be cited. There is no requirement to cite these works unless the editor has indicated otherwise.

Please review your reference list to ensure that it is complete and correct. If you have cited papers that have been retracted, please include the rationale for doing so in the manuscript text, or remove these references and replace them with relevant current references. Any changes to the reference list should be mentioned in the rebuttal letter that accompanies your revised manuscript. If you need to cite a retracted article, indicate the article’s retracted status in the References list and also include a citation and full reference for the retraction notice.

[Note: HTML markup is below. Please do not edit.]

Reviewers' comments:

Reviewer's Responses to Questions

**Comments to the Author**

1. Does the manuscript provide a valid rationale for the proposed study, with clearly identified and justified research questions?

The research question outlined is expected to address a valid academic problem or topic and contribute to the base of knowledge in the field.

Reviewer #1: Yes

**********

2. Is the protocol technically sound and planned in a manner that will lead to a meaningful outcome and allow testing the stated hypotheses?

The manuscript should describe the methods in sufficient detail to prevent undisclosed flexibility in the experimental procedure or analysis pipeline, including sufficient outcome-neutral conditions (e.g. necessary controls, absence of floor or ceiling effects) to test the proposed hypotheses and a statistical power analysis where applicable. As there may be aspects of the methodology and analysis which can only be refined once the work is undertaken, authors should outline potential assumptions and explicitly describe what aspects of the proposed analyses, if any, are exploratory.

Reviewer #1: Yes

**********

3. Is the methodology feasible and described in sufficient detail to allow the work to be replicable?

Descriptions of methods and materials in the protocol should be reported in sufficient detail for another researcher to reproduce all experiments and analyses. The protocol should describe the appropriate controls, sample size calculations, and replication needed to ensure that the data are robust and reproducible.

Reviewer #1: Yes

**********

4. Have the authors described where all data underlying the findings will be made available when the study is complete?

The PLOS Data policy requires authors to make all data underlying the findings described in their manuscript fully available without restriction, with rare exception, at the time of publication. The data should be provided as part of the manuscript or its supporting information, or deposited to a public repository. For example, in addition to summary statistics, the data points behind means, medians and variance measures should be available. If there are restrictions on publicly sharing data—e.g. participant privacy or use of data from a third party—those must be specified.

Reviewer #1: Yes

**********

5. Is the manuscript presented in an intelligible fashion and written in standard English?

PLOS ONE does not copyedit accepted manuscripts, so the language in submitted articles must be clear, correct, and unambiguous. Any typographical or grammatical errors should be corrected at revision, so please note any specific errors here.

Reviewer #1: Yes

**********

6. Review Comments to the Author

Please use the space provided to explain your answers to the questions above and, if applicable, provide comments about issues authors must address before this protocol can be accepted for publication. You may also include additional comments for the author, including concerns about research or publication ethics.

You may also provide optional suggestions and comments to authors that they might find helpful in planning their study.

(Please upload your review as an attachment if it exceeds 20,000 characters)

Reviewer #1: This work by Andreas C. Themistocleous and colleagues is a scientifically interesting and clinically relevant study protocol addressing an important aspect in neuropathic pain research. The manuscript proposes a large-scale observational study combining deep clinical phenotyping with microneurography, quantitative sensory testing, nerve conduction studies, skin biopsy, and genetic/transcriptomic analyses to investigate the relationship between nociceptor excitability and chronic neuropathic pain. The protocol is ambitious, timely, and potentially valuable for mechanism-based patient stratification. For the type of results that can be obtained and generated, it can be at the same time a valuable basic, translational and clinical research study protocol.

A major strength of the protocol is its use of microneurography, which the authors appropriately present as the only technique that directly records from human nociceptors in vivo. The integration of this method with multidomain phenotyping is a clear asset and could generate highly informative mechanistic data. The manuscript is also important for acknowledging practical limitations such as tolerability of extended microneurography and the observational nature of the study.

I have two main suggestions that would strengthen the manuscript further.

Comment #1. Clarify the primary endpoint and the principal analytical unit more explicitly.

The manuscript states that the primary outcome will compare microneurography measures of axonal excitability and axonal properties between participants with and without neuropathic pain. However, the protocol then presents both participant-level and fibre-level primary outcome comparisons. Later, in the bias mitigation section, the authors state that mixed-effects models will be used to account for within-participant variability due to multiple receptive fields/fibres. These are all reasonable elements, but the current presentation leaves some ambiguity regarding what the true primary endpoint is, what the primary unit of inference will be, and how multiplicity and dependence between fibres from the same participant will be handled in confirmatory analyses. A clearer hierarchy of primary / secondary outcomes and analytical levels would improve methodological transparency.

Comment #2. The manuscript would benefit from a final round of language editing. There are a few noticeable typographical or grammatical issues in the current text, which do not undermine the science but do affect presentation. For example: “due a lesion”, “Neary 10%”, “a 11-point numerical scale in to quantify”, “activity dependant”, “Ethical approved”, and a broken sentence: “sensitisation.to mechanical and thermal stimulation”

Overall recommendation:

This is a strong and potentially impactful protocol. I support publication after minor revision aimed at improving precision in the presentation of the primary analysis.

**********

7. PLOS authors have the option to publish the peer review history of their article (what does this mean?). If published, this will include your full peer review and any attached files.

If you choose “no”, your identity will remain anonymous but your review may still be made public.

**Do you want your identity to be public for this peer review?**  For information about this choice, including consent withdrawal, please see our Privacy Policy.

Reviewer #1: No

**********

[NOTE: If reviewer comments were submitted as an attachment file, they will be attached to this email and accessible via the submission site. Please log into your account, locate the manuscript record, and check for the action link "View Attachments". If this link does not appear, there are no attachment files.]

To ensure your figures meet our technical requirements, please review our figure guidelines: https://journals.plos.org/plosone/s/figures

You may also use PLOS’s free figure tool, NAAS, to help you prepare publication quality figures: https://journals.plos.org/plosone/s/figures#loc-tools-for-figure-preparation.

NAAS will assess whether your figures meet our technical requirements by comparing each figure against our figure specifications.

# Author comment: Author response to Decision Letter 1

**DOI:** 10.1371/journal.pone.0349407.r002

28 Apr 2026

Dear Editorial team

Thank you for the opportunity to submit revisions for our manuscript, “Defining the link between peripheral neuronal activity and neuropathic pain: observational study protocol to investigate in vivo neurophysiological properties of nociceptors in patients with chronic neuropathic pain”.

Our responses to the comments are highlighted in red:

Comment #1. Clarify the primary endpoint and the principal analytical unit more explicitly.

The manuscript states that the primary outcome will compare microneurography measures of axonal excitability and axonal properties between participants with and without neuropathic pain. However, the protocol then presents both participant-level and fibre-level primary outcome comparisons. Later, in the bias mitigation section, the authors state that mixed-effects models will be used to account for within-participant variability due to multiple receptive fields/fibres. These are all reasonable elements, but the current presentation leaves some ambiguity regarding what the true primary endpoint is, what the primary unit of inference will be, and how multiplicity and dependence between fibres from the same participant will be handled in confirmatory analyses. A clearer hierarchy of primary / secondary outcomes and analytical levels would improve methodological transparency.

We thank the reviewer for this thoughtful and constructive comment. We agree that clearer specification of the primary endpoint, the principal unit of inference, and the analytical hierarchy strengthens the methodological transparency of the protocol.

We clarify that the primary endpoint is the between-group difference in microneurography-derived measures of axonal excitability and axonal properties between participants with and without neuropathic pain, as originally intended. And that the primary unit of inference is at the participant level. Even when including individual fibre-level data in mixed models we specifically include the participant as the random effect, this way we use the fibre as the unit of observation and maintain the participant as the unit of inference across the study.

The text on page 18 now reads “Comparison of mean values and distributions of axonal excitability and axonal property measures at a participant level for individual nociceptive fibres (for example, spontaneous activity, multiple spikes, mechanical and thermal sensitisation, conduction velocity, and activity-dependent slowing).”

On page 19, we have included the following text to clarify how multiplicity and dependence between fibres from the same participant will be handled in confirmatory analyses:

“Data from individual nociceptive fibres will be analysed with explicit consideration of clustering within participants. Where appropriate, fibre-level measurements will be summarised at the participant level (for example, by averaging) to provide an independent observation per participant for primary analyses. In addition, mixed-effects models will be used, with participant included as a random effect, to account for within-participant variability. Multiplicity arising from multiple outcomes will be addressed using appropriate multiple-comparison adjustments (for example, Bonferroni or false discovery rate correction).

This dual approach allows us to perform conservative primary analyses at the participant level by averaging, while also utilising more sophisticated modelling through a mixed-effects model that retains the detail of the individual fibre data while correcting for the dependence between fibres from the same participant. The mixed-effects model will bridge the participant (unit of inference) and individual fibre (unit of observation) without the statistical error of treating fibres from the same person as independent data points.”

Comment #2. The manuscript would benefit from a final round of language editing. There are a few noticeable typographical or grammatical issues in the current text, which do not undermine the science but do affect presentation. For example: “due a lesion”, “Neary 10%”, “a 11-point numerical scale in to quantify”, “activity dependant”, “Ethical approved”, and a broken sentence: “sensitisation.to mechanical and thermal stimulation”

Thank you for highlighting these errors. We completed the necessary edits.

Attachment Submitted filename: Response to Reviewers.docx

# Editor report: Decision Letter 1

**DOI:** 10.1371/journal.pone.0349407.r003

**Authors:** Armando Almeida, Armando Almeida

30 Apr 2026

Defining the link between peripheral neuronal activity and neuropathic pain: observational study protocol to investigate in vivo neurophysiological properties of nociceptors in patients with chronic neuropathic pain

PONE-D-26-06203R1

Dear Dr. Themistocleous,

We’re pleased to inform you that your manuscript has been judged scientifically suitable for publication and will be formally accepted for publication once it meets all outstanding technical requirements.

Within one week, you’ll receive an e-mail detailing the required amendments. When these have been addressed, you’ll receive a formal acceptance letter and your manuscript will be scheduled for publication.

An invoice will be generated when your article is formally accepted. Please note, if your institution has a publishing partnership with PLOS and your article meets the relevant criteria, all or part of your publication costs will be covered. Please make sure your user information is up-to-date by logging into Editorial Manager at Editorial Manager® and clicking the ‘Update My Information' link at the top of the page. For questions related to billing, please contact billing support.

If your institution or institutions have a press office, please notify them about your upcoming paper to help maximize its impact. If they’ll be preparing press materials, please inform our press team as soon as possible -- no later than 48 hours after receiving the formal acceptance. Your manuscript will remain under strict press embargo until 2 pm Eastern Time on the date of publication. For more information, please contact onepress@plos.org.

Kind regards,

Armando Almeida

Academic Editor

PLOS One

Additional Editor Comments (optional):

Reviewers' comments:

# Editor report: Acceptance letter

**DOI:** 10.1371/journal.pone.0349407.r004

**Authors:** Armando Almeida, Armando Almeida

PONE-D-26-06203R1

PLOS One

Dear Dr. Themistocleous,

I'm pleased to inform you that your manuscript has been deemed suitable for publication in PLOS One. Congratulations! Your manuscript is now being handed over to our production team.

At this stage, our production department will prepare your paper for publication. This includes ensuring the following:

* All references, tables, and figures are properly cited

* All relevant supporting information is included in the manuscript submission,

* There are no issues that prevent the paper from being properly typeset

You will receive further instructions from the production team, including instructions on how to review your proof when it is ready. Please keep in mind that we are working through a large volume of accepted articles, so please give us a few days to review your paper and let you know the next and final steps.

Lastly, if your institution or institutions have a press office, please let them know about your upcoming paper now to help maximize its impact. If they'll be preparing press materials, please inform our press team within the next 48 hours. Your manuscript will remain under strict press embargo until 2 pm Eastern Time on the date of publication. For more information, please contact onepress@plos.org.

You will receive an invoice from PLOS for your publication fee after your manuscript has reached the completed accept phase. If you receive an email requesting payment before acceptance or for any other service, this may be a phishing scheme. Learn how to identify phishing emails and protect your accounts at https://explore.plos.org/phishing.

If we can help with anything else, please email us at customercare@plos.org.

Thank you for submitting your work to PLOS ONE and supporting open access.

Kind regards,

PLOS ONE Editorial Office Staff

on behalf of

Prof. Armando Almeida

Academic Editor

PLOS One
